# Maladaptive Neural Synchrony in Tinnitus: Origin and Restoration

**DOI:** 10.3389/fneur.2015.00029

**Published:** 2015-02-17

**Authors:** Jos J. Eggermont, Peter A. Tass

**Affiliations:** ^1^Department of Physiology and Pharmacology, University of Calgary, Calgary, AB, Canada; ^2^Department of Psychology, University of Calgary, Calgary, AB, Canada; ^3^Institute of Neuroscience and Medicine – Neuromodulation (INM-7), Research Center Jülich, Jülich, Germany; ^4^Department of Neurosurgery, Stanford University, Stanford, CA, USA; ^5^Department of Neuromodulation, University of Cologne, Cologne, Germany

**Keywords:** tinnitus, neural plasticity, neural synchrony, brain rhythms, coordinated reset

## Abstract

Tinnitus is the conscious perception of sound heard in the absence of physical sound sources external or internal to the body, reflected in aberrant neural synchrony of spontaneous or resting-state brain activity. Neural synchrony is generated by the nearly simultaneous firing of individual neurons, of the synchronization of membrane-potential changes in local neural groups as reflected in the local field potentials, resulting in the presence of oscillatory brain waves in the EEG. Noise-induced hearing loss, often resulting in tinnitus, causes a reorganization of the tonotopic map in auditory cortex and increased spontaneous firing rates and neural synchrony. Spontaneous brain rhythms rely on neural synchrony. Abnormal neural synchrony in tinnitus appears to be confined to specific frequency bands of brain rhythms. Increases in delta-band activity are generated by deafferented/deprived neuronal networks resulting from hearing loss. Coordinated reset (CR) stimulation was developed in order to specifically counteract such abnormal neuronal synchrony by desynchronization. The goal of acoustic CR neuromodulation is to desynchronize tinnitus-related abnormal delta-band oscillations. CR neuromodulation does not require permanent stimulus delivery in order to achieve long-lasting desynchronization or even a full-blown anti-kindling but may have cumulative effects, i.e., the effect of different CR epochs separated by pauses may accumulate. Unlike other approaches, acoustic CR neuromodulation does not intend to reduce tinnitus-related neuronal activity by employing lateral inhibition. The potential efficacy of acoustic CR modulation was shown in a clinical proof of concept trial, where effects achieved in 12 weeks of treatment delivered 4–6 h/day persisted through a preplanned 4-week therapy pause and showed sustained long-term effects after 10 months of therapy, leading to 75% responders.

## Introduction

Tinnitus is the conscious perception of sound heard in the absence of physical sound sources external or internal to the body. Sound perceived from physical sound sources inside the body such as blood flow and middle ear muscle twitching is generally called “objective tinnitus”; we will not deal with those here. About 10–15% of adults experience tinnitus. Tinnitus is generally ignited by hearing loss, and very often by noise-induced hearing loss (NIHL), but most chronic tinnitus is of central origin; that is, it is in the brain and not generated in the ear. An illustrative example is found in patients with one-sided deafness, which often experience tinnitus referred to that ear, yet the tinnitus typically subsides when that ear is stimulated via a cochlear implant. As stated in Eggermont (1): “the localization of tinnitus to one or both ears is thus likely attributable to a phantom sensation and is not unlike that related to sensations or pain experienced after losing a digit or, more severely, a limb. Itch or pain in a no-longer-existing part of the body is truly annoying and so is tinnitus. The pitch of tinnitus corresponds, when there is a hearing loss, to the frequency region of that hearing loss. In case of low-frequency hearing loss, the tinnitus is low pitched, but in high-frequency NIHL the tinnitus has a high-pitched ringing or hissing sound.” More than a century ago, MacNaughton-Jones (2) who studied 260 cases of tinnitus described the sounds of tinnitus as follows:

The following were the noises I have recorded as complained of by patients. The sound resembling buzzing; sea roaring; trees agitated; singing of kettle; bellows; bee humming; noise of shell; horse out of breath, puffing; thumping noise; continual beating; crackling sounds in the head; train; vibration of a metal; whistle of an engine; steam engine puffing; furnace blowing; constant hammering; rushing water; sea waves; drumming; rain falling; booming; railway whistling; distant thunder; chirping of birds; kettle boiling; waterfall; mill wheel; music; bells.

As in a true phantom sensation, the brain “hears” the sound of the missing frequencies in one ear, both ears, or inside the head, but describing how it sounds appears to be very personal and typically referred to with known external sounds. Electrophysiological (3) and functional imaging measurements in humans and animals suggested the following neural correlates of tinnitus in the auditory system: increased neural synchrony, reorganization of tonotopic maps, and increased spontaneous firing rates (SFRs) in the auditory system. Tinnitus is likely the result of maladaptive plasticity of the central nervous system. The central nervous system wants to restore its evoked neural activity levels that had been lowered by the hearing loss. This is done by increasing the efficacy (or gain) of its synapses. But this gain also affects the SFR, which occurs in the absence of a physical sound source, and will then generally increase (4). This is interpreted as sound and called tinnitus. A puzzling aspect is that only 30% of people with clinical hearing-loss experience tinnitus (5), so there must be other purely central nervous system aspects that promote or allow the perception of tinnitus. One of these may be neural synchrony reflected in brain rhythms.

### Synchrony in the brain

Neural synchrony is a reflection of the nearly simultaneous firing of individual neurons (micro synchrony), of the synchronization of membrane-potential changes in local neural groups as reflected in the local field potentials (LFP; mesosynchrony), or of the presence of oscillatory brain waves in the EEG (macro synchrony). In the current literature, micro- and mesosynchrony has only been measured in animals, whereas macro synchrony is limited to human data. A general review on large-scale integration in the brain and the role of neural synchrony therein is presented by Varela et al. (6).

#### Microscopic synchrony

On statistical and geometrical grounds (7), the probability that two cortical neurons with the axonal arborization of one neuron overlapping the dendritic tree of the other neuron make no synaptic contacts is very high (~0.9), and the probability of making just one contact is thus relatively low (~0.1). Assuming independence of contacting (synapse formation), the probability for making two (~0.01) and more than two contacts are therefore negligibly small. Thus in the cortical system of pyramidal cell to pyramidal cell connections, the influence of one neuron onto another (micro synchrony) is very weak. The effect of increased interneuron synchrony is generally a more efficient excitation of downstream neurons, especially at the cortical level, where synaptic strengths are weak. Abeles (8) first drew attention to the power of synchronously arriving inputs to activate cortical neurons. The consequence of correlated neural input is that the amplitude of the post-synaptic potentials (PSPs) resulting from these inputs will be proportional to the number of inputs, whereas the PSP amplitude from asynchronously arriving inputs is only proportional to the square root of their number. The synaptic connection strengths, and firing rates for thalamic cells combined, are potentially a factor 3–4 higher than those of cortico-cortical cells (9). Then, even only 30 synchronously firing specific afferents [the putative number converging on a cortical cell (10)], which produce compound PSPs that are 3–4 times larger than for cortico-cortical cells and effectively produce 90–120 amplitude units of PSP, would still be as effective as 9000 asynchronously firing non-specific cortico-cortical inputs that produce √ (9000) ≈ 95 amplitude units of PSP. Given that a typical cortical cell receives ~10,000 inputs, the few synchronous ones are potentially as effective as all the other asynchronous ones together.

#### Mesoscopic synchrony

The relationship of fast LFP oscillations with neuronal spike discharges consistently shows that these discharges are generally occurring at negative going deflections of the depth-recorded LFPs in auditory cortex (11, 12). The spontaneous firing of single neurons is also highly correlated with the LFP as reflected in spike-triggered averaging of the LFP (13). Initially, excitatory PSPs were considered the most important source of LFPs (14), other sources such as inhibitory PSPs, other subthreshold membrane-potential oscillations, and action potential afterpotentials also contribute significantly to the LFP (15). Low-frequency (<10 Hz) oscillations in the LFP may reflect global neuromodulatory inputs (16). Thus, pyramidal neurons may be the strongest contributors to the LFP due to their dipole size, geometry, and parallel-oriented dendritic trees. Gamma-band (40–80 Hz) rhythms, likely originate in cortical microcircuits consisting of pyramidal cells and interneurons (15). They also stated that: “interneurons play an important role in generating the underlying gamma-oscillations. They act as (oscillation) generators via rhythmic inhibition of pyramidal neurons and their synchronized inhibitory synaptic potentials also contribute significantly to the membrane-potential oscillations on pyramidal neurons (17, 18).” GABAergic neurons dominate the generation of gamma-band oscillations, whereas excitatory connections determine the amplitude, duration, and long-range synchronization of these oscillations (19). In order for the LFP to be recorded, a large number of neurons should synchronously change their membrane potential. Thus, the amplitude of the LFP represents mesosynchrony at the population membrane-potential level.

Eggermont et al. (12) made multi-electrode array recordings of spike and local field potential activity from primary auditory cortex of 12 normal hearing, ketamine-anesthetized cats. They compared spike and LFP characteristic frequency (CF) gradients and their cross-correlation distances, and concluded that: “The CF gradient for spike-based FTCs was about twice that for 2–40 Hz-filtered LFP-based FTCs, indicating greatly reduced frequency selectivity for LFPs. For spontaneous LFP and spike activity, we evaluated 1373 pair correlations for pairs with >200 spikes in 900 s per electrode. Peak correlation-coefficient space constants were similar for the 2–40 Hz-filtered LFP (5.5 mm) and the 16–40 Hz LFP (7.4 mm), whereas for spike-pair correlations it was about half that, at 3.2 mm. Comparing spike pairs with 2–40 Hz (and 16–40 Hz) LFP-pair correlations showed that about 16% (9%) of the variance in the spike-pair correlations could be explained from LFP-pair-correlations recorded on the same electrodes within the same electrode array. This larger correlation distance combined with the reduced CF gradient and much reduced frequency selectivity suggests that LFPs are not a substitute for spike activity in primary auditory cortex.”

#### Macroscopic synchrony

The generation of detectable spontaneous oscillatory activity in the EEG requires synchronous activity of large numbers of neurons and in addition spatial alignment of the underlying LFP dipoles, which largely restricts the contributing elements to cortical pyramidal cells. The fast gamma- and beta-band (20–40 Hz) frequencies in the EEG spectrum are occurring in the context of the slower alpha (8–14 Hz), theta (4–7 Hz), and delta (1–4 Hz) bands (6, 20). The mutual influence between specific nuclei of the thalamus and the cortex occurs across different frequencies such as the alpha-range around 10 Hz (21, 22), which are involved in setting and resetting the cycles of excitatory PSPs on pyramidal cells. The theta band in limbic structures appears during memory consolidation. These slower rhythms, including the delta band, could provide the temporal frame (20, 23) within which the beta and gamma rhythms operate. In general, auditory stimuli increase the frequency of thalamo-cortical rhythms to gamma-band rates and reduce the amplitudes of alpha-range frequencies. In the deafferented state, however, the thalamo-cortical activity decreases in frequency to those of the theta band (16).

The frequency of network oscillations in cortex as reflected in the EEG ranges from slow oscillations in the delta and theta ranges to fast oscillations in the gamma and ultrafast (90–200 Hz) ranges. Gamma oscillations in particular have been proposed as reference signals for sensory binding of features into a coherent percept (24). Network oscillations require periodic and synchronized neuronal activity. If several neurons fire both regularly and synchronously, the synaptic activation pattern results in a rhythmic LFP (17). If large numbers of neurons are activated simultaneously, the corresponding currents then add up in phase and become so large that they can be detected at the scalp (25). Albeit that the strength of EEG, or MEG signals depends on the degree of synchrony of the local field potentials and spiking activity, confounding variables make it difficult to draw conclusions on this synchrony by considering only the power of the various brain rhythms (26).

Spontaneous EEG or MEG activity relies on neural synchrony; without synchrony, the EEG would just be noise. Neural synchrony appears to be confined to several frequency bands. In general, there is a correlation between the frequency of these brain rhythms and the distance over which synchronization is observed. Higher frequencies (gamma-band) tend to show synchronization over shorter distances than that in the lower-frequency theta- and alpha-band. The latter, when occurring over the *temporal* lobe, is typically referred to as tau rhythm (27). Consistent slower occipital α rhythms during wakefulness may indicate pathology (28), e.g., manifesting itself as depression (29) and neurogenic pain (30). A decrease in alpha-rhythm frequency toward that of the theta band has been suggested to result from an abnormal disfacilitation in the thalamus and has been called “thalamic dysrhythmia” of Llinas et al. (30).

### Synchrony changes following hearing loss

#### Microscopic synchrony in animal auditory cortex

Noise-induced hearing-loss causes a reorganization of the tonotopic map in cat primary auditory cortex and increases both SFR and neural synchrony (31). The peak cross-correlation coefficient (*R*) for spike-pair firings was increased immediately after a traumatizing noise exposure (32). They found that the change in *R* only affected the recordings more than one octave above the trauma tone frequency (TTF). Previously, Eggermont and Komiya (33) had shown that the “reorganized part” of AI (units with a pre-trauma CF above the TTF) showed an increased proportion of significant correlation peaks. Seki and Eggermont (34) and later Noreña and Eggermont (31) recorded from between 2 weeks and 4 months after noise exposure, and found a significant increase in *R* (by 40%) in the reorganized parts of AI. These frequency-dependent changes in *R* in permanent NIHL are then consistent with the acute ones seen in Noreña and Eggermont (32). It was noted that immediately after the trauma the frequency tuning of neurons shifted dominantly toward the TTF accompanied by an increase in response areas (35). An increase in overlap of their response areas should then be paralleled by an increase in their common “synchronized” inputs from the thalamus. A few hours after the trauma, *R* was further increased, and nearly independent of the pre-trauma CF difference. These changes were significantly correlated with those in the SFR. However, the increase in *R* immediately after the trauma occurred without changes in SFR.

These changes in tonotopic maps, SFR, and increased neural synchrony (a commonly occurring triad) could be prevented by rearing the noise-trauma cats in an enhanced acoustic environment (EAE) that compensates for the reduced firing activity in the high-frequency neurons as a result of the NIHL (31, 36). This suggests that the changes in tonotopic maps, SFR, and neural synchrony are likely all due to an across-frequency imbalance of neural output activity from the cochlea. NIHL is not the only condition that may cause increases in neural synchrony and increased SFR. They also found these after exposing adult cats to a 4–20 kHz non-traumatizing sound for >5 months at a level of 80 dB SPL peak equivalent. No changes in ABR threshold were found, but most neurons in AI ceased to respond to frequencies between 4 and 20 kHz. Characteristic frequencies of neurons would normally be in the 4–20 kHz range, based on their location in AI, now all responded to either frequencies >20 kHz, <4 kHz, or to both. For the neurons outside the reorganized range, the SFRs were increased and their pair correlations showed greatly increased cross-correlation coefficients (37).

Engineer et al. (38) exposed rats for 1 h to a 115-dB SPL, octave-band noise centered at 16 kHz. This resulted in about 15–20 dB permanent hearing loss between 4 and 32 kHz (as measured by ABR) at 11 weeks post-trauma. At this time, there were clear indications of tonotopic map reorganization in AI. This contrasts with the absence of map reorganization in cats, which was only found when the hearing loss was more than 25 dB (39, 40). They also found that the average SFR in AI and the cross-correlation coefficient for spontaneous multi-unit activity recorded at hearing-loss sites in AI were significantly increased. Out of the 28 noise-exposed rats used in this study, 18 were significantly impaired in their ability to detect a gap, as evidenced by increased gap-startle responses, in narrowband noise centered on 8 or 10 kHz. This was considered an indication for the presence of tinnitus with a pitch in the 8–10 kHz region. In these 18 rats, changes in the tonotopic map, but not increased SFR or synchronization, were significantly correlated with the degree of gap-startle enhancement in noise-exposed rats. This suggests that if we accept the validity of the gap-startle to indicate tinnitus, that neither microsynchrony measure was related to tinnitus (41).

Macroscopic synchrony has so far not been studied in animals with hearing loss or tinnitus.

#### Macroscopic synchrony in tinnitus patients

The spontaneous MEG in a group of individuals with tinnitus was characterized by a marked reduction in tau-band power together with an enhancement in delta-band power as compared to normal-hearing controls (42). Tinnitus-related distress was correlated with this reduced tau- and increased delta-band activity, particularly in right temporal and left frontal areas (43). This could be related to the often comorbid depression in tinnitus patients. Reduction of tau-rhythm power is a normal cortical reaction to sound presentation (44) just as visual stimulation (eyes open) reduces the occipital alpha rhythm. Thus, the results of Weisz et al. (43) could suggest that a decrease of tau-rhythm power in tinnitus patients may be related to the tinnitus perception itself. Kahlbrock and Weisz (45) found that reduction of tinnitus loudness following residual inhibition resulted in a reduction of delta activity. Because delta activity is potentially generated by input-deprived neuronal networks (46), residual inhibition may result from a short-duration re-establishment of balance between excitatory and inhibitory neuronal assemblies.

Gamma-band oscillations have been linked to conscious sensory perception and positive symptoms in a variety of neurological disorders (46). Weisz et al. (47) examined gamma-band activity during brief periods of marked enhancement of delta-band activity [see Ref. (43)]. They found that both control and tinnitus groups showed significant increases in gamma-band activity after the onset of delta waves, but this was more prominent in the tinnitus subject than in controls. The hemispheric lateralization of the gamma-band activity did correlate with the lateralization of the perceived tinnitus. Ashton et al. (48) observed in eight patients with tinnitus unilateral foci of high-frequency gamma (>40–80 Hz) activity over the auditory cortex. These hotspots were not present in subjects without tinnitus.

In unilateral tinnitus patients, the source strength of the resting-state gamma-band rhythm in the auditory cortex contralateral to the tinnitus ear showed a strong positive correlation with the tinnitus loudness (49). This reflects a similar sound-evoked activation of the contralateral auditory cortex as observed in normal hearing. Recently, the reciprocal relationship between reduced tau-activity and increased gamma activity in tinnitus patients was further strengthened (50). We have seen that depending on the frequency band of the EEG oscillations, local or more global neural synchrony is implicated. Especially, the long-range (several cm) synchrony coupling appears to be affected in tinnitus patients. In particular, coupling in the 9–12 Hz frequency band was reduced and that in the 45–54 Hz frequency band was increased (51). They found that: “In patients with a tinnitus history of <4 years, the left temporal cortex was predominant in the gamma network whereas in patients with tinnitus duration of more than 4 years, the gamma network was more widely distributed including more frontal and parietal regions.” Schlee et al. (52) postulated that the hyperactivity of the temporal cortices in tinnitus patients is integrated in a global network of long-range cortical connectivity. This long-range cortical activity was related to the degree of the tinnitus distress.

A different set of findings was presented by Adjamian et al. (53) studied the effect of hearing loss and tinnitus separately and in combination on various brain rhythms using resting-state MEG. They found that delta-band activity was significantly higher in the “tinnitus with hearing loss” group compared to the “no tinnitus with normal hearing” group. The delta activity was reduced, but not significantly, when tinnitus was masked. They suggested that “a combination of {tinnitus] and hearing loss is necessary for the observed increase in delta-band activity.” In contrast, theta and alpha activity was not significantly different for the presence of tinnitus in the hearing-loss group. Furthermore, in this study, gamma-band activity was not correlated with either tinnitus or hearing loss.

The majority of human studies were conducted in patients with chronic tinnitus (see above). However, an MEG study in amateur rock musicians with a short-term tinnitus immediately after band practice revealed that the transient tinnitus was accompanied by temporary hearing loss and increased gamma activity in the right cortex in 13 out of 14 cases (54). The subject group was non-homogenous concerning their tinnitus laterality: 8 out of 14 subjects reported a bilateral tinnitus, 3 subjects perceived their tinnitus on the right side, and the remaining three patients on the left side. In general, Ortmann and coworkers were not able to disentangle whether the increased gamma-band activity was due to the hearing loss and/or the tinnitus percept (54). In particular, no evidence was provided for gamma being directly related to the tinnitus percept (54). Furthermore, the pattern of abnormal oscillatory power observed in patients with transient tinnitus (right-lateralized increase of gamma) differed from that observed in chronic tinnitus sufferers [increased slow-wave and gamma activity combined with reduced alpha, see Ref. (43, 47)]. Given these results, one might speculate whether the functional role of gamma is different for acute vs. chronic tinnitus, whether gamma might be related to hearing-loss rather than to the tinnitus percept, or whether different gamma-associated processes might take place (54). Given the subjects’ non-homogenous tinnitus laterality, it is particularly difficult to sort out the differential relationship between hearing impairment vs. tinnitus percept and gamma.

### Animal vs. human data

Comparison of animal data with results obtained in humans with MEG, EEG, or LFP measurements has to be drawn carefully. In animal experiments and in human studies, neuronal dynamics is studied on different scales. While in animal cortical studies, typically neuronal spikes or spike-bursts are analyzed (31, 32, 55), in human studies, scalp EEG and MEG oscillations are investigated, which are generated by neocortical post-synaptic potential rhythms. Those attributed to primary auditory cortex may partially reflect those of LFPs recorded in animal auditory cortex. Also most data from animals were obtained under anesthesia. Voss et al. (56) found that the intravenously applied anesthetic etomidate significantly increased both LFP amplitude and slope of the population spike in neocortical slice. This suggested increased neuronal synchrony within the recording area, estimated to have a diameter of 300 μm. A sleeping brain also shows higher neural correlations that extend over larger distances than in an awake brain or a brain that is processing information [evidence reviewed in Ref. (57)]. One could say that the neurons in a sleeping brain are part of several large assemblies. In contrast, one may assume that in an information processing brain, regardless whether it is under light anesthesia or not, neurons with different response properties will not very likely be doing the same thing at the same time and thus will show less synchronized firings (58).

In all these comparisons, between animal and human neural synchrony it cannot be stressed enough that hearing loss may have a larger effect on brain rhythms than tinnitus, as has only recently been demonstrated (53, 59–61).

In human research papers, “neuronal synchronization” reflects coincident firing in a large population of neurons that furthermore need to have their current dipoles oriented in parallel. This temporal and spatial alignment occurs in cortical pyramidal cells and allows large-amplitude oscillations in EEG and MEG signals. In contrast, cross-correlation analysis in animals is typically performed for single unit and/or multi-unit activity (55, 62). Thus, the simultaneous recording and analysis of spikes and LFPs (only practical in animals) may provide complementary information about the overall network activity. The finding of different frequency tuning for LFPs and spikes has been reported for primary auditory cortex of the cat (12, 63).

The problem of relating measurements at different levels is highlighted by the observation that evidence linking gamma-band oscillations to spike-pair cross-correlations is not completely consistent. Cardin et al. (64) found that light-driven (optogenetic) activation of fast-spiking interneurons at varied frequencies (8–200 Hz) in barrel cortex selectively amplified gamma oscillations while activation of pyramidal neurons amplified only lower-frequency oscillations (e.g., the delta band), revealing a cell-type-specific double dissociation. Given that the cortical spike–spike correlations measured in the animal models of tinnitus were between putative pyramidal cells, it is not straightforward to associate the increased correlation strength for auditory cortical pyramidal cells in animal models with increased power of gamma-band oscillations in humans with tinnitus. However, other studies make the case for pyramidal cell pair correlations and gamma power in the cortices. Denker et al. (65) related synchrony at the level of microscopic neural-spike firing and at the mesoscopic LFP activity in motor cortex. It appeared that coincident pyramidal cell spikes were better phase locked to the LFP than could be predicted by the locking of the individual spikes with the LFP. Overall pair-wise spike synchrony could not be explained by their firing rates. Denker et al. (65) suggested that “precise spike synchrony constitutes a major temporally and spatially organized component of the LFP.” How this is reflected in macroscopic brain activity remains to be explored.

### Role of neural synchrony in tinnitus perception

Although, in the animal auditory cortex, neural cross-correlation studies are not performed routinely, one can summarize the findings as showing a significant increase in neural synchrony between neural spiking in separate cortical areas during stimulation compared with spontaneous firing. The strength of neural synchrony in the cat auditory cortex (66) did not depend on the supra-threshold stimulus level. Thus, the abrupt change in neural synchrony between sub- and supra-threshold conditions may help, together with the increase in firing rate, in signaling the presence of sound. Neural synchrony has also been implicated in signaling the presence of a continuous tone in the absence of any difference in firing rate from the spontaneous condition (67, 68). Thus, neural synchrony may signal both a change from silence to sound and the presence of an ongoing stimulus. The latter may be important in the perception of tinnitus. What does this mean in relation to macro synchrony in the EEG?

Synchronized gamma-band activity in general is proposed to bind sensory events into one coherent conscious percept (24). Tinnitus, as a constant auditory phantom percept is potentially dependent on persistent gamma-band activity in the auditory cortex (49). In normal hearing there is a sound level-dependent activation of the primary auditory cortex in humans as measured with evoked potentials and fMRI (69). Gross et al. (70) showed a close association between induced gamma activity and the perception of pain. Single cell recordings for close-to-threshold somatosensory stimuli suggested that stimulus level is coded in the primary somatosensory cortex whereas the resulting conscious percept is coded in the prefrontal cortex (71). Following the above-mentioned ideas, Van der Loo et al. (49) hypothesized that if tinnitus is a symptom of thalamo-cortical dysrhythmia (46), and if there is a sound intensity-dependent activation of the primary auditory cortex, spontaneous gamma-band oscillations at the level of the primary auditory cortex should correlate with subjective reports of tinnitus loudness in patients with unilateral tinnitus. Van der Loo et al. (49) found that the gamma-band oscillations in auditory cortex were not related to the conscious perception of tinnitus. Thus, “Auditory phantom percepts thus show similar sound level-dependent activation of the contralateral auditory cortex as observed in normal audition. In view of recent consciousness models and tinnitus network models, these results suggest that tinnitus loudness is coded by gamma-band activity in the contralateral auditory cortex but might not, by itself, be responsible for tinnitus perception.” Related to this, Eggermont (41) stated that: “for a sensory stimulus to be consciously perceived, activation of the early sensory areas is a prerequisite but is not sufficient (72). Even in the absence of sensory inputs, cortical and thalamic neurons can show structured patterns of ongoing spontaneous activity (73). These observations are also reflected in an implementation of the neural workspace model (74) in which ascending brain stem nuclei (e.g., cholinergic among others) send globally depolarizing neuromodulatory signals to a thalamic and cortical hierarchy.”

### Resting state brain networks

Tinnitus is aberrant spontaneous or resting-state brain activity interpreted as sound. An exploration of recent findings on resting-state brain activity and its spatial correlations may shed light on some findings in tinnitus patients.

#### Correlated spontaneous activity in the brain

Spontaneous blood oxygen level-dependent (BOLD) activity is not random noise, but reflects a specific organization of activity in the resting human brain (75). Fox et al. (76) identified two widely distributed brain networks on the basis of either spontaneous correlations within each network or negative correlations in activity between networks. These networks consisted of regions that were involved in task-related activations and other of regions corresponding to task-related deactivations. Thus, the same type of correlation between brain areas was present during spontaneous activity as well as task-related activity. These resting-state functional connectivity measures are based on correlations in slow (<0.1 Hz) spontaneous fluctuations in the BOLD signal.

A promising and potentially related electrophysiological correlate of spontaneous BOLD fluctuations is the slow (<0.1 Hz) voltage fluctuation that has been observed with DC-coupled EEG recording (77). These slow rhythms are in turn correlated with the power of higher frequency bands (20). The “nesting” of higher frequency rhythms within these slow fluctuations may be an important principle in brain organization. Much animal work has focused on slow (<1 Hz) fluctuations in electrical membrane potential, also referred to as up and down states (78). Whether these fluctuations are related to those in the BOLD signal needs further investigation.

Eggermont (79) was the first to establish that spike-pair correlations in anesthetized cat primary auditory cortex (AI) during spontaneous activity were predictive of those during stimulus-evoked activity. In that paper, it was shown that the cross-correlation coefficients for 71 dual-electrode pairs with clearly visible correlation peaks were similar for stimulus (after shift-predictor correction) and spontaneous conditions. A paired *t*-test showed that the spontaneous cross-correlation-coefficient values were on average only 0.004 larger than during stimulation. Under stimulus conditions, the peak of the neural correlogram was generally narrower than under spontaneous firing conditions. This was the case in 62–87% of correlograms, and depended on the stimulus type. The adequacy of a particular stimulus played a role as well; in general the narrower correlograms were obtained for the more effective stimuli. Most likely, the cause for this narrowing of the correlogram was a more synchronous arrival of thalamic afferent activity at the cortical cells, so that especially the delayed coincidences resulting from di- and polysynaptic interactions that make up the base of the correlogram peak were better synchronized. Thus, spontaneous functional connectivity is very similar to that under stimulus conditions. Nevertheless, a hierarchical cluster analysis showed that neuron clusters typically consisted of several neuron pairs with ≤1 mm distance, and changed their borders somewhat between spontaneous and stimulus conditions (62). Tsodyks et al. (80) using optical imaging, which reflects local field potential activity, demonstrated that spontaneous activity in the cat visual cortex is highly coordinated across large assemblies. This was reflected in the spontaneous discharges of individual neurons that were temporally locked to the activation of other cells with similar orientation preferences. The patterns of coherence in spontaneous LFP activity can span distances of several millimeters (12, 81), indicating that specific long-range interactions are possible even in the absence of visual or auditory input. These data make it likely that spontaneous activity may contain structured information and therefore plays an important role in cortical function. Pathological changes in these network correlations may be underlying tinnitus.

#### Network correlations in tinnitus

Maudoux et al. (82, 83) tested 13 patients with chronic tinnitus and 15 age-matched healthy controls with a 3 T MRI scanner during resting condition (i.e., eyes closed, no task performance). Connectivity was investigated using independent component analysis (ICA). They found that tinnitus and control groups showed different connectivity patterns. In the control group, these patterns formed two distinct negatively correlated networks. The first included the auditory cortices and the insula. The second one comprised frontoparietal and anterior cingulate cortices, brainstem, amygdala, basal ganglia/nucleus accumbens (NAc), and parahippocampal regions. The tinnitus group showed an increased functional connectivity between auditory cortical areas and the left parahippocampal region. Connectivity in non-auditory regions such as brainstem, basal ganglia/NAc, cerebellum, parahippocampal, right prefrontal, parietal, and sensorimotor areas was also increased in tinnitus subjects. Thus, in tinnitus patients, there were changes in the connectivity of functional networks that serve attention, memory, and emotion.

An increase in functional connectivity between auditory and parahippocampal regions in tinnitus was found by Vanneste et al. (84) who used resting-state EEG measurements. Based on resting-state electrical brain activity of tinnitus patients and controls, they reported an increased gamma-frequency activity in the parahippocampal area. They also found an increase in connectivity between parahippocampal regions and auditory cortical areas in tinnitus patients compared to control subjects. High- and low-distressed tinnitus patients showed different degrees of activation of the left middle frontal gyrus, supporting a putative fronto-parietal-cingulate network, which may be more active in highly distressed tinnitus patients (85). This middle frontal gyrus had previously been linked to the perception of tinnitus (43) and more recently also to tinnitus distress (86) Two decades ago, Jastreboff (87) had already suggested that the prefrontal cortex was a region for integrating sensory and emotional characteristics of tinnitus.

In bothersome tinnitus, the functional connectivity between auditory and occipital/visual cortex showed reciprocal negative correlations (88). They stated that: “Negative correlations indicate that when BOLD response magnitudes increased in auditory or visual cortex they decreased in the linked visual or auditory cortex, suggesting reciprocally phase-reversed activity between functionally connected locations in tinnitus. Both groups showed similar connectivity with positive correlations within the auditory network. Primary visual cortex in tinnitus showed extensive negative correlations in the ventral attention temporoparietal junction and in the inferior frontal gyrus and rostral insula – executive control network components. Rostral insula and inferior frontal gyrus activity in tinnitus patients also showed greater negative correlations with occipital cortex. This implies that in bothersome tinnitus, there is a dissociation between activity in auditory cortex and visual, attention, and control networks.” In a follow-up study, Wineland et al. (89) found that among non-bothersome tinnitus patients, the tinnitus percept did not alter the functional connectivity of the auditory cortex or other key cortical regions.

Schmidt et al. (59) investigated auditory, dorsal attention, and default mode networks in adults with tinnitus (TIN) and hearing loss and normal hearing without tinnitus (NH) controls using resting-state fMRI. They used ICA and found “increased connectivity between the left parahippocampus and the auditory resting-state network in the TIN group when compared to NH controls. Similarly, there was also an increased correlation between the right parahippocampus and the dorsal attention network when compared to HL controls. Other group differences in this attention network included decreased correlations between the seed regions and the right supramarginal gyrus in TIN patients when compared to HL controls. In the default mode network, there was a strong decrease in correlation between the seed regions and the precuneus when compared to both control groups. The findings of this study identify specific alterations in the connectivity of the default mode, dorsal attention, and auditory resting-state networks due to tinnitus.”

Davies et al. (90) also used ICA to identify coherent patterns arising from spontaneous brain signals within the resting-state data. Their study design “carefully matched participant groups for possible confounds, such as hearing status. Twelve patients (7 male, 5 female; mean age 66 years) all with chronic constant tinnitus and 11 controls (8 male, 3 female; mean age 68 years) took part. No significant differences were found in auditory network connectivity between groups after correcting for multiple statistical comparisons in the analysis. This contradicts previous findings reporting reduced auditory network connectivity; albeit at a less stringent statistical threshold.” In the Davies et al. (90) study, the auditory network connectivity was not affected by chronic tinnitus.

### Electrical kindling and synchrony

Anticonvulsant drugs have often been tried to alleviate tinnitus based on the assumption that hypersynchrony underlies both epilepsy and tinnitus. Kindling refers to a highly persistent modification of brain functioning in response to repeated application of initially sub-convulsant electrical stimulation, typically in the limbic system, which results in the development of epileptiform activity (91). The effect of electrical kindling could also be demonstrated by stimulation of primary auditory cortex (92). Kindling by stimulating AI twice daily for 1 s with 60 biphasic pulses of 200–400 μA peak value for 2–3 weeks, caused approximately two-thirds of the animals to reach a fully generalized convulsive state in 40 stimulation sessions. After the animals reached this state, multi-unit recordings were obtained under ketamine anesthesia from AI contralateral to the kindled site. The spontaneous firing pair peak cross-correlation coefficient showed an increase of ~40%. The tonotopic map in AI was dramatically changed and showed a large area that was tuned to frequencies approximating CF of the electrical stimulation site in contralateral cortex.

Electrical kindling appears to cause similarities to the effects of traumatic noise exposure. As Eggermont (93) wrote: “The role of GABA and glutamate receptors in kindling and NIHL is also comparable. Kindling is associated with a loss in GABAergic inhibition (94). It has also been hypothesized that kindling stimulation in cortex produces an accumulation of glutamate. This may trigger altered NMDA channel functions and long-lasting changes in synaptic efficacy of long-range (several mm distance) horizontal connections (95, 96). In cat AI, horizontal connections generally follow a course parallel to the iso-frequency contours (97) but others project in directions orthogonal to the iso-frequency contours and may even extend as far as anterior and posterior auditory fields (98). Strengthening of the horizontal connections after kindling may explain the larger percentage of double-tuned frequency-tuning curves that we observed (92). A similar emergence of multi-tuned neurons was found after long-term exposure to a spectrally (5–20 kHz) EAE that did not cause hearing loss but still resulted in a dramatic cortical reorganization and increased neural synchrony (37). If these horizontal connections become stronger than those of the specific thalamic inputs, they could elicit an outward migration of glutamate hyperactivity and subsequent synaptic changes. This process would likely stop when all the neurons in the stimulated cortical area (and its projection region) were combined into one large neural assembly.”

One could thus entertain the notion that correlated neural activity may result in cortical map reorganization. Cortico-cortical connections are very often found between neurons with characteristic frequencies differing by more than one octave (99). Consequently, these neurons may have non-overlapping receptive fields but still can have sizeable peak cross-correlations as shown by Tomita and Eggermont (100). Correlated neural activity and heterotopic neural interconnections are potentially the substrates for cortical reorganization. As increased neural synchrony and tonotopic map reorganization go hand in hand, this suggests that these are important driving forces underlying tinnitus (93).

### Coordinated reset mechanisms

#### Basic principles and features of coordinated reset neuromodulation

In neuronal populations, changes of patterns of activity and synaptic connectivity are strongly connected (101) by spike timing-dependent plasticity (STDP) (102, 103): synaptic weights (i.e., the strength of synaptic connections) are up- or down-regulated subject to the relative timing of pre- and post-synaptic spikes. Networks with STDP are generically multistable (104–107): concerning their fast neuronal dynamics as well as their slow synaptic dynamics (i.e., connectivity pattern) qualitatively different attractors (i.e., stable regimes) coexist. Stable synchronized states characterized by strong mean synaptic strength exist side by side with stable desynchronized states characterized by weak mean synaptic strength. In computational studies, it was shown that networks with STDP can be shifted from one attractor to another by appropriate stimulation, in this way giving rise to stimulation effects persisting after cessation of stimulation (104–107).

Based on a computational approach (108), coordinated reset (CR) stimulation (109, 110) was developed in order to specifically counteract abnormal neuronal synchrony by desynchronization. CR stimulation means to stimulate different neuronal subpopulations of a target population through different stimulation sites sequentially at different times in order to reset the phases of the different subpopulations equidistantly in time (109, 110). For electrical CR stimulation, brief high-frequency pulse trains are typically used as resetting stimuli. CR stimulation causes a desynchronization and, hence, a reduction of the rate of coincidences (104, 109, 110). In this way, a network with STDP can be shifted from a pathological model state with abnormally strong synapses to a desynchronized state with weaker synapses (104–107). Put otherwise, CR neuromodulation causes anti-kindling, i.e., the network gets reshaped and unlearns up-regulated synchrony.

Coordinated reset was successfully applied to deep brain stimulation (DBS). In a pre-clinical study, electrical CR neuromodulation was applied to the subthalamic nucleus (STN) in parkinsonian non-human primates via depth electrodes for only 2 h/day 5 days running (111). Assessments of motor function demonstrated acute as well as sustained long-lasting therapeutic after effects for up to 30 days. In a human proof of concept study in six externalized parkinsonian patients, electrical CR neuromodulation delivered to the STN 3 days running with a daily dose of up to 2 h × 2 h stimulation caused a significant and cumulative reduction of beta oscillations in the STN together with a correlated significant improvement of motor function (112). In addition, CR-induced long-lasting desynchronization was demonstrated experimentally in rat hippocampal slice with epileptiform activity induced by magnesium withdrawal (113).

Coordinated reset neuromodulation does not require permanent stimulus delivery in order to achieve long-lasting desynchronization or even a full-blown anti-kindling. Rather, as shown computationally, CR may have cumulative effects, i.e., the effect of different CR epochs separated by pauses may accumulate (114). This was verified, e.g., in a study on electrical CR neuromodulation of the STN in externalized parkinsonian patients (112).

Apart from desynchronization, CR may have additional effects. According to computational studies, CR need not change the firing rates (109, 110). However, if the CR frequency (i.e., the repetition rate at which CR stimuli are delivered) strongly deviates from the mean firing/bursting rate, changes of the firing rate are to be expected. Note that changes of the firing rate are not in contradiction to desynchronizing effect. For instance, computationally, it was shown that even an activating CR stimulation delivered to a population of mostly inactive neurons increases the mean firing rate without any increase of the global synchrony (115). From a theoretical point of view, desynchronization can be achieved at minimal stimulation intensities if the CR frequency is adapted to the mean firing/bursting rate (109, 110).

A phase reset is a universal dynamical phenomenon, which can be achieved by a variety of stimulation setups as shown in a large number of modeling and experimental studies, e.g., by hyperpolarizing or depolarizing electrical pulses (116–119), excitatory or inhibitory PSPs (120–124), and sensory stimulation (125–128) as well as transcranial magnetic stimulation (129). Accordingly, in a computational study, desynchronization and anti-kindling were robustly achieved in populations of spiking and bursting model neurons coupled by excitatory and inhibitory adaptive synapses irrespective of whether CR was delivered by direct electrical stimulation or indirect, synaptically mediated excitatory and/or inhibitory stimulation (130). Consequently, the invasive CR approach was extended to non-invasive, in particular, acoustic CR neuromodulation. To this end, based on the tonotopic organization of the central auditory system, instead of applying electrical stimulation bursts to different brain sites, CR was realized by delivered tones of different pitch (131, 132).

The particular spatiotemporal pattern of stimulus delivery is key to the effects of multisite stimulation (106, 133). For instance, intuitively one might expect that independent noise, i.e., noise delivered to each neuron in an uncorrelated manner, would be extremely powerful for desynchronizing a population of synchronized neurons. However, in a computational study, it was shown that for oscillatory neural populations with STDP quite the opposite is true. In fact, the strength of the synapses increases with increasing noise intensity and reaches a maximum in a resonance-like manner known from stochastic or coherence resonances (133). This holds for both excitatory and inhibitory synaptic input. In other words, over a wide range of the noise intensity, the uncorrelated noise basically stabilizes the abnormally up-regulated synaptic connections. This finding might be relevant in the context of the mechanism of action of noises and maskers.

#### Acoustic CR neuromodulation

**Figure 1:**
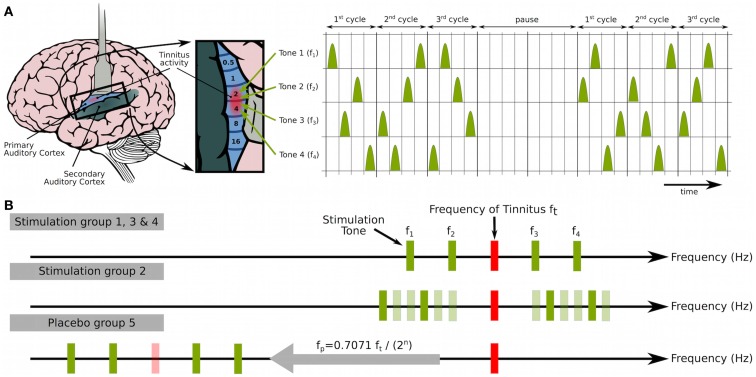
**Acoustic coordinated reset (CR) neuromodulation**. **(A)** CR neuromodulation means to deliver phase resetting stimuli to neuronal subpopulations in a spatiotemporally coordinated manner in order to induce desynchronization and eventually anti-kindling (104, 109, 110): employing the tonotopic organization of the primary auditory cortex [left, brain adapted from Chittka and Brockmann (135) with kind permission of the authors] short sinusoidal tones of different frequencies were grouped within approximately one octave around the tinnitus frequency *f*_t_ (*f*_1_ = 0.77 *f*_t_, *f*_4_ = 1,40 *f*_t_) to induce a soft reset (134) of different parts of the synchronized tinnitus focus, respectively. Three CR cycles, each containing a randomized sequence of four tones (left), were followed by two silent cycles (“pause”). That pattern was repeated periodically [compare (104, 109, 110, 115)]. **(B)** The proof of concept study by Tass et al. (132) comprised four stimulation groups (G1–G4) and one placebo group (G5), where G2 served as active control group. Patients in groups G1, G3, and G4 were treated with acoustic CR neuromodulation, i.e., with four tones (top, *f*_1_–*f*_4_) grouped around the tinnitus frequency (*f*_t_). In all patients, *f*_t_ was assessed with a pure tone matching. G3 differs only in repetition rate *F* (i.e., the inverse of the duration of a cycle), which was adapted to the individual EEG δ-band peak at each visit. According to computational studies, continuous online adaptation of *F* should be beneficial (109, 110). In all other groups, the repetition rate *F* was set to 1.5 Hz to target delta oscillations (109, 110). Stimulation dosage was 4–6 h/day in G1–G3 and 1 h/day in G4 and G5. For G2 in each CR cycle, a random selection of four tones (dark green: active) was taken out of 12 (middle, *f*_1_–*f*_12_) surrounding *f*_t_. For placebo stimulation (bottom, G5), a similar pattern as for G1 was used, but with down-shifted stimulation frequency *f*_p_ [*f*_p_ = 0.7071 *f*_t_/(2*^n^*), and *f*_p_ within (300 Hz, 600 Hz)] to ensure stimulation outside the synchronized tinnitus focus. Figure from Tass et al. (132) with kind permission by the authors. Copyright by Forschungszentrum Jülich GmbH.

The goal of acoustic CR neuromodulation is to desynchronize tinnitus-related abnormal delta-band oscillations in order to finally cause anti-kindling (132). Taking advantage of the tonotopic organization of the central auditory system, e.g., the primary auditory cortex (Figure 1A), brief sinusoidal tones of different frequencies (pitch) are grouped within approximately one octave around the audiologically determined tinnitus frequency to induce a soft reset (134) of different neuronal subpopulations comprised in the synchronized tinnitus focus, respectively. A soft reset means that a phase reset is achieved by means of a repetitive stimulus delivery, e.g., after three CR cycles (Figure 1A). Properly calibrated CR stimulation tones are perceived by patients as just supra threshold and equally loud (132).

Acoustic CR neuromodulation aims at separately stimulating different subpopulations of the abnormally synchronized neuronal population in order to induce phase resets of the corresponding neuronal subpopulations. If the CR tones are unfavorably aligned, e.g., in the case of a small-size synchronized population and a comparatively widespread alignment of CR tones, some of the CR tones might impact the related subpopulation only indirectly, via lateral inhibition. Nevertheless, CR might still exert its specific effect. Indeed, computationally in populations of spiking as well as bursting neurons, it was shown that even a randomly mixed excitatory–inhibitory CR leads to anti-kindling (130). To this end, a randomly selected fraction of the neuronal population received inhibitory stimuli, and the rest of the ensemble was stimulated excitatorily. Irrespective of the portion of neurons receiving inhibitory stimulation (ranging from 0 to 100%), robust anti-kindling was achieved.

Unlike other approaches, acoustic CR neuromodulation does not intend to reduce tinnitus-related neuronal activity by employing lateral inhibition. For instance, based on the notion of lateral inhibition, notched music was designed to induce short-term plastic changes (136), and tailor-made notched music was tested as a means to counteract tinnitus symptoms (137). However, how a cortical population impacts on another, abnormally synchronized cortical population via lateral inhibition may decisively depend on the activity pattern of the pre-synaptic population generating the lateral inhibition. In this context, let us recall computational studies on the effects of different types of multisite stimulation delivered via inhibitory synapses to populations of firing or bursting neurons with STDP. In fact, different inhibitory stimulation paradigms may actually have totally different effects as opposed to inhibition: while uncorrelated inhibitory noise stabilizes abnormally up-regulated synaptic connections (133), inhibitory CR neuromodulation causes anti-kindling (130), whereas permanent high-frequency stimulation blocks neuronal firing without any long-lasting effects after cessation of stimulation (107).

#### Phase-amplitude coupling of gamma to delta

Gamma-band oscillations have been linked to conscious perception and positive symptoms in a variety of neurological disorders (46). In unilateral tinnitus patients, source analysis of resting-state gamma-band oscillations showed a strong positive correlation of the source strength in the contralateral auditory cortex with tinnitus loudness (49). Tinnitus thus shows similar loudness-dependent activation of the contralateral auditory cortex as observed in normal hearing. It is thus important to link gamma-band activity to the delta-band activity that CR modulation aims to disrupt. Young and Eggermont (20) described the link as:

While three principal properties of an oscillation (amplitude, frequency, and phase) can combine and generate many types of coupling, only amplitude–amplitude, amplitude–phase and phase–phase relationships have been actively explored in the brain. Amplitude–amplitude coupling is perhaps the easiest to compute; however, its functional meaning is not clear at all. Within a single record, amplitude–amplitude coupling is suggestive of a general increase in excitability modulation, but does not offer any insights to how field potential oscillations at certain frequencies contribute to the function, with or without each other. Between areas, amplitude–amplitude coupling can be indicative of two recorded areas belonging to the same functional group, modulated by a common source or directly modulated activities in the respective areas. However, this measure cannot provide conclusive evidence of functional interaction between the areas and again offers no insight to how the two areas cooperate in a behavioral setting.

In an EEG study in patients with chronic subjective tinnitus, abnormalities of the cross-frequency coupling (CFC) were found both within and between areas (138). Tinnitus patients showed an increased phase-amplitude CFC within the auditory cortex and the dorsolateral prefrontal regions between the delta and theta phase and the gamma amplitude. In addition, the gamma-band amplitude in the auditory and dorsolateral prefrontal regions was modulated by the theta phase in the anterior cingulate region modulated.

### Clinical findings related to acoustic CR neuromodulation

Safety and efficacy of acoustic CR neuromodulation were studied in a prospective, single blind, placebo-controlled, randomized trial in 63 patients with chronic tonal tinnitus and hearing loss of up to 50 dB (132). The CR treatment was safe and well-tolerated and caused a highly significant reduction of tinnitus loudness and symptoms according to visual analog scale (VAS) and TF scores (132). “Tinnitus-Fragebogen” [TF (139)] is the German adaptation of Hallam’s (140) tinnitus questionnaire (TQ). To differentiate between acute and long-lasting effects of stimulation, VAS scores were measured in the off-stimulation condition, i.e., at least 2.5 h after turning off CR neuromodulation, and subsequently in the on-stimulation condition, i.e., 15 min after onset of CR neuromodulation, while TF scores were measured off-stimulation. CR treatment was delivered for 12 weeks with a portable acoustic device and comfortable earphones with a subsequent off-stimulation 4-week period (without treatment and any consultation or the like) to investigate theoretically predicted (104, 131) lasting effects of acoustic CR neuromodulation. Subsequently, patients were offered an optional open-label long-term extension (LTE) period of 24 weeks duration. During the 12-weeks treatment period, acoustic CR neuromodulation was delivered at different dosages and repetition rates and was compared to a placebo stimulation, which did not have any significant effects, and a noisy variant of CR neuromodulation, which had masker-like acute, but no lasting effects and served as active control (Figure 1).

Effects gained with 4–6 h acoustic CR neuromodulation/day during the 12-week treatment phase subsisted through the subsequent 4-week therapy pause at a slightly reduced, but significant level (instead of a complete wash-out) and promptly regained the reduction in VAS after resuming the CR therapy after 16 weeks. In contrast, only in a minority of patients noisers and maskers have tinnitus-reducing after effects, and the latter last only seconds to minutes (141, 142). A comparison of the active arms (G1–G4 in Figure 1) in the study by Tass et al. (132) revealed that CR therapy was more efficacious at a daily dose of 4–6 h as opposed to 1 h. The noisy CR neuromodulation (G2 in Figure 1) also had pronounced acute effects, but no lasting stimulation effect, comparably to the short-term residual inhibition of noises or maskers (143). The lack of long-term effects of noisy CR neuromodulation might be due to the different tones being located close to each other (see Figure 1), so that in a relevant number of stimulation cycles a separate stimulation of neuronal subpopulation with neighboring tones, as required for CR, was impossible. The net effect of the noisy CR stimulation (G2) might, hence, be a suppression of tinnitus-related activity mediated by lateral inhibition. Placebo stimulation (G5) caused limited and non-significant changes in VAS and TF scores, which were of comparable size as, e.g., effects of inpatient treatment including cognitive behavioral therapy (144). Presumably placebo stimulation was ineffective since it was delivered far apart from the synchronized tinnitus focus (see Figure 1).

During the LTE, all patients were offered the treatment with acoustic CR neuromodulation with 4–6 h/day (G1). The responder analysis according to Goebel et al. (144) of the TF data obtained at the end of the LTE, i.e., at 40 weeks, revealed 40% winners (defined by a TF improvement of at least 15 points), 35% responders (TF improvement of 6–14 points), 23% non-responders (TF unchanged, i.e., ±5 points), and 2% losers (defined by a TF worsening of at least 6 points). Taken together, at the end of the LTE, 75% of the patients were winners or responders, and their TF was reduced by 50% on average. The response to CR treatment was influenced neither by the duration of tinnitus nor by the severity of tinnitus at the beginning of therapy. Clinical effects gained with acoustic CR neuromodulation were both statistically (132) and clinically (145, 146) significant.

Apart from clinical effects, also electrophysiological effects were studied by means of EEG recordings and subsequent data analysis. In the CR treatment group (G1 in Figure 1A) after 12 weeks, the temporal spectral power in the delta band was significantly decreased, whereas the alpha power was significantly increased (132). To perform a more involved analysis, requiring a sufficiently large number of patients, good responders were selected with a reliable-change-index (RCI) (147) that was applied to improvements of TF scores (132). The vast majority of good responders were patients treated with acoustic CR neuromodulation for 4–6 h/day (G1 and G3 in Figure 1A) accompanied by a minority of patients treated with acoustic CR neuromodulation delivered for only 1 h/day (148). None of the patients from the active control group (G2) or the placebo group (G5) made it into the group of good responders. For this group of good responders, a number of EEG analyses were performed and compared to a healthy control group: EEG power analysis reflecting the extent of neuronal synchrony (132, 148, 149), effective connectivity analysis within frequency bands (148), CFC analysis (138), subgroup analysis of therapy induced pitch change-related changes of EEG power and connectivity (150).

### Specific effects of CR neuromodulation

#### CR-induced reduction of EEG spectral power

**Figure 2:**
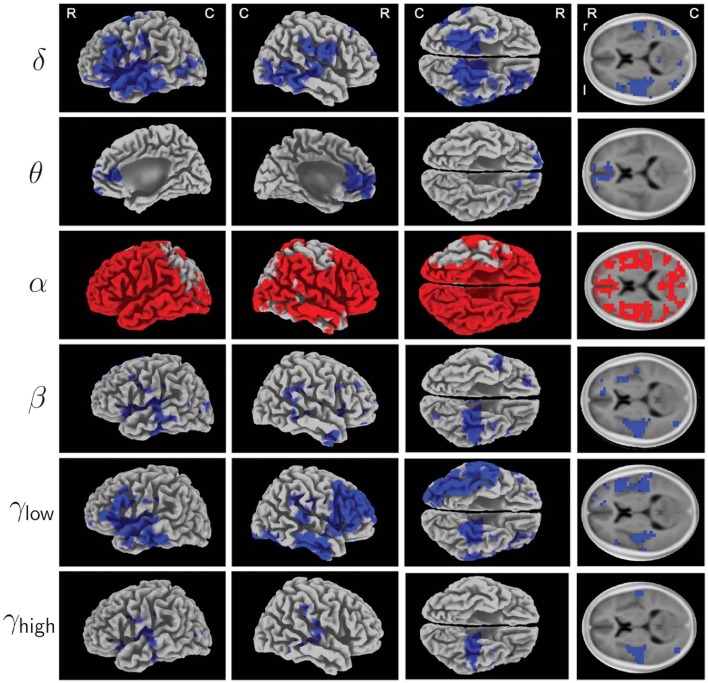
**Electrophysiological effects of acoustic CR neuromodulation studied by Tass et al. (132): 3D mapping of treatment induced changes in spectral power of oscillatory EEG activity (baseline compared to 12 weeks, recorded in off-stimulation condition)**. To increase signal-to-noise ratio, 12 patients with bilateral tinnitus (from G1, G3, and G4, see Figure 1) were selected using a reliable-change-index (RCI) (147) applied to improvements of TF scores. Statistical non-parametric maps from sLORETA (152) provide localization of changes of δ (1–4 Hz), θ (4–8 Hz), α (8–12 Hz), β (12–30 Hz), γ_low_ (30–48 Hz), and γ_high_ (52–90 Hz) spectral power. Results were superimposed onto a three-dimensional brain (first three columns) and onto a horizontal brain section (right column) of a standard anatomical template. Significantly decreased spectral power after acoustic CR neuromodulation compared to baseline is labeled blue, increased spectral power is labeled red (corrected, *p* < 0.05). Abbreviations: *R*, rostral; C, caudal; r, right; l, left. Figure from Tass et al. (132) with kind permission by the authors. Copyright by Forschungszentrum Jülich GmbH.

Acoustic CR neuromodulation selectively counteracted tinnitus-related EEG power abnormalities (132): statistical non-parametric (151) 3D standardized low-resolution brain electromagnetic tomography (sLORETA) maps (152) revealed that 12 weeks of CR treatment significantly reduced delta and gamma power in a network containing several auditory and non-auditory areas (blue voxels in Figure 2). Delta (1–4 Hz) power was primarily reduced in temporal and prefrontal areas, for instance, in the primary and secondary auditory cortices. In frontal areas and the anterior cingulated area, theta (4–8 Hz) power was significantly reduced [Brodmann Area (BA) 32]. Beta (12–30 Hz) power significantly decreased in temporal areas, in particular, in the superior temporal gyrus (bilateral, BA 41, 42). In the temporal and frontal cortex, comprising larger areas in the right prefrontal cortex, low gamma power (30–48 Hz) was significantly reduced, whereas high gamma power (52–90 Hz) was significantly reduced in the temporal cortex, particularly strongly on the left side and most pronounced in the superior temporal gyrus (BA 41). In addition, CR caused a widespread bilateral increase in alpha (8–12 Hz) power, most pronounced in temporal areas and in the entire prefrontal cortex (red voxels in Figure 2).

These changes were confirmed by a different analysis, basically employing empirical mode decomposition instead of bandpass filtering (148). Comparing these data with a healthy control group, it was shown that the CR-induced changes of the amplitude of brain rhythms actually normalized the oscillatory power in the tinnitus-associated network of brain areas, in particular, in temporal regions (149). Furthermore, a positive association between changes in tinnitus severity and the normalization of delta and gamma power in temporal, parietal, and cingulate cortical areas was found.

#### CR-induced reduction of abnormal effective connectivity

Tinnitus patients showed pronounced abnormalities in effective connectivity as assessed by applying a combination of empirical mode decomposition and partial directed coherence (153) to current sources reconstructed from EEG recordings. Comparing untreated good responders and a healthy control group revealed a tinnitus-related significant increase of the effective connectivity between the left primary auditory cortex and the posterior cingulate cortex in the gamma and delta bands, along with a significant decrease of the effective connectivity between the right primary auditory cortex and the dorsolateral prefrontal cortex in the α band. CR had a marked impact on effective connectivity. Hardly, any pathological interaction persisted for the 12 weeks CR treatment, so that, in fact, the patterns of effective connectivity of good responders and healthy controls became statistically indistinguishable. An additional dynamic causal modeling (DCM) analysis (154) revealed the specific action of CR therapy, which counteracted the imbalance of excitation and inhibition. In particular, caused by CR the strength of the inhibitory connections between auditory cortices and the dorsolateral prefrontal cortex significantly increased, whereas the strength of the excitatory connection between posterior cingulate cortex and primary auditory cortex significantly decreased (148).

#### CR-induced reduction of cross-frequency coupling

Tinnitus patients showed pronounced abnormalities in CFC. In tinnitus patients, phase-amplitude CFC was increased within the auditory cortex and the dorsolateral prefrontal regions between the delta and theta phase and the gamma amplitude (138). In addition, the theta phase in the anterior cingulate region modulated the gamma amplitude in the auditory and dorsolateral prefrontal regions. Interestingly, in a computational study, it was shown that a slow rhythmic input into an oscillatory neuronal population may cause a modulation of the amplitude of a fast oscillation over a wide range of input intensities (155). Furthermore, a reduction of tinnitus severity after acoustic CR therapy was associated with a partial normalization of abnormal CFC, where changes in CFC were significantly modulated by treatment-induced changes in tinnitus pitch (138).

#### CR-induced tinnitus-pitch changes

Acoustic CR neuromodulation significantly reduced the tinnitus frequency as assessed by pure tone matching (132). To study whether and how clinical as well as electrophysiological changes relate to CR-induced tinnitus frequency changes, sLORETA (152) was applied to EEG recordings from two subgroups of good responders (with a sustained CR-induced relief of tinnitus symptoms) with and without tinnitus-pitch change, i.e., two subgroups of the group of good responders [from Tass et al. (132)] used for the EEG studies mentioned above (150): from a clinical standpoint, a significant correlation was found between absolute changes of VAS loudness and VAS annoyance scores on the one hand with the modulus of the tinnitus-pitch change on the other hand. These clinical differences were reflected by electrophysiological disparities: unlike in patients with weak or no pitch change in patients with pronounced tinnitus-pitch change, gamma power decreased significantly stronger in left temporal cortex (BA 22, 42), left frontal cortex (BA 4, 6), and right parietal cortex (BA 40) as well as right frontal cortex (BA 9, 46). The differential decrease of γ power was combined with a significantly stronger enhancement of α power in the right and left anterior cingulate cortex (BA 32, 24). In addition, in patients with pronounced pitch change, the functional connectivity in the gamma-band between the right dorsolateral prefrontal cortex (BA 46) and the right anterior cingulate cortex (BA 32) was significantly weaker after the 12-week CR treatment phase. Overall, these results suggest that CR significantly reduces tinnitus-associated auditory binding in a network of brain areas involved in pitch processing.

The clinical and electrophysiological findings mentioned in this section (132, 148, 149) are in accordance with theoretical predictions about long-lasting CR-induced desynchronization (104, 131). In addition, these studies show that acoustic CR neuromodulation also counteracts abnormal patterns of effective connectivity (148) and CFC (138). Interestingly, acoustic CR neuromodulation typically causes a pronounced shift of the tinnitus pitch (132, 150). On the one hand, this is indicative of central plastic mechanisms being employed by the CR treatment. On the other hand, the CR-induced tinnitus-pitch change is clinically relevant since it requires regular recalibration of the CR tones in the course of the treatment. The treatment-induced shift of the tinnitus pitch has not been predicted theoretically. This might be due to the clinically used (approximately logarithmic) alignment of the CR tones not sufficiently reflecting the equidistantly spaced setup in the computational model and/or the computational models lacking relevant features, e.g., a gradient of the lateral inhibition (156).

## Discussion and Outlook

Many studies indicate the important role of abnormal neuronal synchrony in the pathophysiology of subjective tinnitus. However, despite the growing body of evidence, there are still significant open questions. For instance, it is important to separate the effects of hearing loss from the effects of tinnitus perception on the emergence of abnormal neuronal synchrony. In their pioneering MEG study, Weisz et al. (43) revealed abnormally increased delta and decreased alpha power in tinnitus patients with high-frequency hearing loss as opposed to a normal-hearing control group. Increased delta power was found in the same brain regions as decreased alpha, similarly to findings obtained during slow-wave sleep (157). Hence, the observed changes in spontaneous brain waves might simply be due to deafferentation, i.e., the high-frequency hearing loss, instead of being specific for tinnitus (43).

There are different ways to cope with this problem. On the one hand, one can use hearing-level-matched controls as done in only a few studies so far, e.g., employing fMRI (158), voxel-based morphometry (60, 61), resting-state fMRI (59), and brainstem auditory evoked potentials (159). In fact, even more sophisticated designs with, e.g., four groups (comprising tinnitus patients with hearing loss, tinnitus patients with clinically normal hearing, no tinnitus with hearing loss, and no tinnitus with clinically normal hearing), as used in an MEG study (53), might further help to reveal the differential contributions of hearing loss and tinnitus to abnormal neuronal synchrony. On the other hand, one can compare the same group of tinnitus patients under different conditions, with tinnitus and with significantly reduced tinnitus, as done in studies employing neurofeedback (160), residual inhibition (45), and acoustic CR neuromodulation (132, 138, 148, 149, 150). Since no significant changes in auditory thresholds were detected pre/post-CR treatment, the pronounced and significant changes in oscillatory power (reflecting neuronal synchrony) (132, 149), effective connectivity (148), and CFC (138) reviewed above can be attributed to different extents of tinnitus symptoms. To further our understanding of hearing loss- and tinnitus percept-related patterns of brain synchrony, one could, e.g., compare group of tinnitus patients pre- and post-clinically effective intervention with a hearing-level-matched control group.

Coordinated reset neuromodulation was developed along the lines of a top down-approach, starting with phase oscillator networks (108–110), later on employing qualitatively different types of spiking and bursting neural networks, coupling topologies, and stimulation modalities (105–107, 114, 131). CR neuromodulation turned out to robustly and effectively induce anti-kindling in these different model networks. The fact that CR works in quite diverse neural networks underlines its robustness, which might be key to particular applications such as DBS, since the latter involves stimulation of quite diverse target structures (161). However, to further develop the CR technology, more detailed levels of description are taken into account. For instance, in the field of DBS, biophysically realistic models for target neurons (162, 163) were incorporated into large-scale networks to further the development of dedicated depth electrodes optimized for CR stimulation (164).

Analogously, in the field of tinnitus promising computational studies are available and might lead to improved model versions serving as testbed for optimizing acoustic CR neuromodulation and acoustic stimulation procedures, in general. For instance, concerning the origin of tinnitus and, in particular, its relationship to hearing loss, different computational studies were performed (4, 165–167).

Hearing loss due to cochlear damage causes an overall decrease of auditory nerve activity along with an increase of the SFRs in the dorsal cochlear nucleus (168). In a computational study, it was shown that a homeostatic plasticity mechanism may give rise to the deafferentation-induced hyperactivity of the cochlear nucleus neurons (4). So far, that model takes into account firing rates rather than synchronization processes. In a spiking neuron model, the origin of tinnitus-related abnormal neuronal synchrony was analyzed (166). It was shown that under the assumption of a deafferentation-induced compensatory change in the connection strengths of the lateral excitatory and inhibitory connections, abnormal neuronal synchrony may emerge.

Apart from synchrony, there might be other collective neuronal activity patterns relevant in the context of tinnitus. For instance, traveling waves of neuronal activity appear to be relevant under different pathological conditions, e.g., in migraine (169), epilepsy (170), and brain injury (171). In the visual system, traveling waves were, e.g., computationally studied in the context of migraine (172, 173) and different types of visual hallucinations related to different pathologies (174–176). Whether in the auditory cortex, traveling waves of neuronal activity might emerge as a consequence of deafferentation was studied by Chrostowski et al. (167). In that study, it was proposed that mediated by homeostatic plasticity deafferentation leads to a down-regulation of lateral inhibition, which in turn in the absence of spontaneous activity may lead to the emergence of traveling waves of excitation. In fact, in that model, spontaneous activity prevents from the emergence of long-range traveling waves, in accordance with the lack of empirical evidence for traveling activity waves in the primary auditory cortex (167).

The proof of concept trial performed by Tass et al. (132) was an exploratory first in man trial, which revealed promising results. Despite limitations (due to differences in baseline score, age, and tinnitus duration between treatment groups), the CR treatment enabled to collect safety information as well as dosage-dependent efficacy data for acoustic CR (with a significant decrease in all three clinical scores at proper dosage) that are mandatory for the design of controlled clinical trials (132). In addition, these results were confirmed in a real life study in 200 patients with chronic subjective tinnitus (177). As a next step, a fully powered trial with confirmatory statistics is required to fully prove efficacy.

As yet, acoustic CR neuromodulation is limited to patients with tonal or tone-like tinnitus, with one or a few dominant tones (132). The results obtained so far indicate that CR treatment outcome essentially relies on a good audiological calibration of the CR tones, comprising pitch matching and loudness matching. However, the reliability of tinnitus-pitch matching is limited due to the high variability of results (178–180). Accordingly, it is necessary to further improve the pitch-matching procedure (181). In addition, currently, studies are being undertaken which aim at establishing an objective, EEG-based calibration procedure for the CR tones with the goal to achieve a calibration procedure that is quicker, more reliable, and might also be applicable to patients with atonal tinnitus.

The applicability of acoustic CR neuromodulation might also be limited by severe hearing loss. In a proof of concept study, the acoustic CR treatment was delivered only to patients who were able to hear the CR tones, with an up to moderate hearing impairment (of up to 50 dB within the frequency band of the stimulation tones) (132). In a real life study, acoustic CR neuromodulation was applied to patients with <60 dB hearing loss for all tested frequencies (125 Hz–8 kHz) (177). In both studies, no correlation between hearing loss and reduced therapeutic outcome was reported (132, 177). To overcome limitations due to hearing impairment, acoustic CR neuromodulation could also be delivered by hearing aids and, ultimately, also through cochlear implants (realizing the CR stimulation algorithm by means of electrical pulses). In addition, specifically designed stimulation and dosage protocols might be beneficial, too. For instance, computationally, it was shown that the spacing principle (182), i.e., improving learning by delivering repeated stimuli spaced by pauses as opposed to delivering a massed stimulus (in a single long stimulation session), may enable effective CR-induced anti-kindling at stimulation intensities that are too weak if CR stimulation were to be delivered permanently (183). Spaced acoustic CR neuromodulation might eventually turn out to be an option, e.g., in case of more severe hearing loss or even a near-total hearing loss in the tinnitus region, since at least in computational studies, it is intriguingly effective even at minimal stimulation intensities (183). Of course, such a development requires sound dose finding, proof of concept and, finally, controlled clinical trials.

## Conflict of Interest Statement

Jos J. Eggermont reports no potential conflicts of interest. Peter A. Tass is employed by Research Center Jülich and works as Consulting Professor at Stanford University. Formerly working with ANM GmbH (Cologne, Germany), shareholder of ANM GmbH. Peter A. Tass is the main inventor of more than 220 patents on different neuromodulation techniques; the assignee of this patent portfolio is Research Center Jülich.
